# Simulation and Experimental Study on the Effect of Superheat on Solidification Microstructure Evolution of Billet in Continuous Casting

**DOI:** 10.3390/ma17030682

**Published:** 2024-01-31

**Authors:** Nan Tian, Guifang Zhang, Peng Yan, Pengchao Li, Zhenhua Feng, Xiaoliang Wang

**Affiliations:** 1 Faculty of Metallurgical and Energy Engineering, Kunming University of Science and Technology, Kunming 650093, China; tian1852558@163.com (N.T.); yanp_km@163.com (P.Y.); 13696392999@163.com (P.L.); fengzhenhua666@126.com (Z.F.); wangxiaoliang@kust.edu.cn (X.W.); 2 Key Laboratory of Clean Metallurgy for Complex Iron Resources in Colleges and Universities of Yunnan Province, Kunming University of Science and Technology, Kunming 650093, China; 3 Linyi Iron and Steel Investment Group Special Steel Co., Ltd., Linyi 276000, China

**Keywords:** square billet, superheat, solidification structure, dendrite growth, numerical simulation

## Abstract

The control of the solidification structure of a casting billet is directly correlated with the quality of steel. Variations in superheat can influence the transition from columnar crystals to equiaxed crystals during the solidification process, subsequently impacting the final solidification structure of the billet. In this study, a model of microstructure evolution during billet solidification was established by combining simulation and experiment, and the dendrite growth microstructure evolution during billet solidification under different superheat was studied. The results show that when the superheat is 60 K, the complete solidification time of the casting billet from the end of the 50 mm section is 252 s, when the superheat is 40 K, the complete solidification time of the casting billet is 250 s, and when the superheat is 20 K, the complete solidification time of the casting billet is 245 s. When the superheat is 20 K, the proportion of the equiaxed crystal region is higher—the highest value is 53.35%—and the average grain radius is 0.84556 mm. The proportion of the equiaxed crystal region decreases with the increase of superheat. When the superheat is 60 K, the proportion of the equiaxed crystal region is the lowest—the lowest value is 46.27%—and the average grain radius is 1.07653 mm. Proper reduction of superheat can obviously reduce the size of equiaxed crystal, expand the area of equiaxed crystal and improve the quality of casting billet.

## 1. Introduction

The microstructure simulation of the solidification process refers to the simulation of the solidification process of castings on the grain scale, through which only a few experiments can be conducted to predict the solidification microstructure and mechanical properties of castings [1,2,3]. With the rapid development of computer simulation technology, numerical simulation technology has become an efficient and convenient means to simulate the solidification process of casting and predict the evolution of the solidification process organization. At present, the numerical simulation methods that can be used to predict the microstructure evolution of casting during solidification, include deterministic method, stochastic method, phase-field method, etc. [4,5,6]. In recent years, the stochastic model closest to crystal growth in the solidification process is mainly based on cellular automation (CA) and its derivative simulation methods. The basic idea is that the primitive cell changes the state of the primitive cell according to the change rules of the cell in the similar field, and affects the change of the next cell, so it constantly evolves. This is similar to the process of metal solidification. Zhong et al. [7] studied the effect of superheating and cooling intensity on the solidification structure and macro-segregation of 6Cr13Mo stainless steel billet. The cooling conditions of the continuous casting billet were determined through numerical simulation. The 6Cr13Mo steel sample was solidified in the thermal simulator under simulated cooling conditions. Sheng et al. [8] established a finite element coupled model of cellular automata and studied the effects of cooling spray state and process conditions on the solidification structure of continuous casting slabs. The model is verified. The solidification structure under different cooling spray schemes, superheat and pouring speed was simulated. Cao et al. [9] introduced fractal dimension and specific surface area to quantitatively describe the overall morphology of the solidified structure of continuous casting billet of GCr15 bearing steel, and calculated the permeability of dendrite channel based on this. The results show that the fractal dimension can describe the self-similar complexity and the specific surface area can describe the coarsening degree of the solidified structure. Zhao et al. [10] adopted a two-dimensional cellular automaton (CA) model and finite element (FE) method to simulate solidification structure formation during continuous casting of beam billet, which represents the growth of columnar and equiaxial dendrites under detailed secondary cooling boundary conditions. An et al. [11] established the CAFE model and SDAS model in order to study the evolutionary behavior of macrostructure and secondary dendrites, respectively, on a 220 × 260 mm^2^ bloom cross-section of GCr15 bearing steel. Based on numerical simulation and experiments, the effects of process parameters on center ECR and SDAS are investigated. Xiao et al. [12] researched the grain growth process of X15CrNiSi20-12 austenitic stainless steel in the process of investment casting by using the cellular automaton finite element (CAFE) model, and studied the effects of shell thickness, shell temperature, pouring temperature and cooling rate on the solidification structure via orthogonal test. The results show that the cooling method has an important effect on the formation of axial crystals. The effect of shell temperature, shell thickness and pouring temperature on the formation of axial crystals is small. Yamazaki et al. [13] used the cellular automata method to simulate the solidification structure of steel during continuous casting, and the influence of electromagnetic stirring was also considered in the model.

Based on the production data of casting billet with a carbon content of 0.20wt.%, the potential influence of continuous casting parameters on the solidification process was systematically studied by using the CAFE model. By combining the actual field statistics, we successfully verified the high accuracy of the numerical simulation model by using the low superheat structure test of continuous casting billet.

Continuous casting superheat refers to the temperature above its equilibrium liquidus before pouring molten metal into a mold in a continuous casting process. The setting of the superheat has an important influence on the whole continuous casting process and the properties of the final continuous casting billet. On the basis of model verification, the effects of different superheat parameters on the solidification structure were studied. This study not only contributes to a comprehensive understanding of the solidification behavior of 20# steel under specific process conditions, but also provides practical guidance for industrial production. Through numerical simulation and experimental verification of the system, we provide a new research idea for the optimization of on-site continuous casting parameters, which is expected to play a positive role in improving production efficiency and product quality. The aim of this research is to provide engineers and decision makers in the relevant fields with strong support to better understand the influence of superheat parameters on the solidification structure of 20# steel, and to flexibly apply this knowledge in actual production. Through this research, we expect to contribute to the technological advancement and process optimization of the steel industry and promote innovation and development in related fields.

## 2. Materials and Methods

### 2.1. Nucleation Model

Based on the theory of continuous nucleation, refs. [14,15] finite element (FE) method was used to solve the differential equation of heat conduction [16], and the governing equation of 3D macroscopic temperature field heat conduction is shown as Equation (1).

(1) $$\frac{\partial }{\partial x}\left(\lambda \frac{\partial T}{\partial x}\right)+\frac{\partial }{\partial y}\left(\lambda \frac{\partial T}{\partial y}\right)+\frac{\partial }{\partial z}\left(\lambda \frac{\partial T}{\partial z}\right)+\rho L\frac{\partial f_{s}}{\partial t}=\rho c\frac{\partial T}{\partial t}$$

In Equation (1), T is the thermodynamic temperature, K; ρ is the density, kg/m^3^; c is the specific heat capacity, J/(kg °C); λ is the thermal conductivity, W/(m·K); and L is the latent heat of solidification, J/g.

In order to more accurately reflect the actual situation and take into account the influence of other solidification conditions on the final grain size and grain shape distribution during solidification, the continuous nucleation model based on Gaussian distribution proposed by GANDIN et al. [17,18] was adopted in this study, assuming that nucleation occurs at a series of different nucleation locations. A continuous rather than discrete distribution function *dn*/*d*Δ*T* was used to describe the change in nucleation density [19], where dn is an increase in grain density caused by an increase in subcooling Δ*T*. The density *n*(Δ*T*) of the grains formed at a certain degree of subcooling Δ*T* can be obtained by integrating the distribution curve [20], as shown in Equation (2).

(2) $$n\left(\Delta T\right)=\int_{0}^{\Delta T}\frac{dn}{d\left(\Delta T\right)}d\left(\Delta T\right)$$

The Gaussian distribution of Equation (2) is used to obtain Equation (3):

(3) $$\frac{dn}{d\left(\Delta T\right)}=\frac{N_{s}}{\sqrt{2\pi }\Delta T_{\sigma }}\exp\left[-\frac{1}{2}{\left(\frac{\Delta T-\Delta T_{N}}{\Delta T_{\sigma }}\right)}^{2}\right]$$

In Equation (3), n(Δ*T*) is the nucleus density at subcooling degree Δ*T*, where Δ*T* = *T_L_* − *T* (*T_L_* is liquidus temperature, K); *N_S_* is the maximum nucleation density; and Δ*T_N_* and Δ*T_σ_* are the maximum nucleation supercooling and standard variance supercooling of the alloy, respectively.

### 2.2. Dendrite Growth Model

The transformation from columnar crystals to equiaxed crystals in dendrite growth represents two distinct conditions of crystal growth morphology during the solidification process of continuous casting billets. These conditions are influenced by a multitude of factors, some of which can induce either dendrite growth or the formation of columnar crystals. The temperature gradient is an essential factor that affects crystal growth morphology. A larger temperature gradient typically promotes dendrite growth, as crystals tend to grow faster on one side under high-temperature gradients, resulting in a branched structure. Conversely, a smaller temperature gradient is more conducive to the formation of equiaxed crystals. Simultaneously, the solidification rate plays a crucial role in determining crystal growth morphology. A faster solidification rate generally favors dendritic structures because rapid solidification leads to increased crystal growth rates and facilitates the formation of dendrites. On the other hand, a slower solidification rate is more favorable for the development of columnar equiaxed crystals.

This paper primarily focuses on investigating how changes in superheat influence dendrite growth within the solidified tissue during continuous casting processes. In conclusion, variations in superheat effectively impact both the temperature gradient and solidification rate within casting billets, thereby significantly influencing dendrite growth under different superheating conditions.

In this study, the KGT model [21,22] was used to calculate the dendrite tip growth rate. The KGT model is a dendrite growth model established by Kurz, Giovanola and Trivedi in 1986 on a steady-state basis [23]. According to the boundary stability criterion [24], Equation (4) can be obtained:

(4) $$V^{2}\frac{\pi^{2}\Gamma }{P^{2}D^{2}}+V\frac{mC_{0}\left(1-k_{0}\right)}{D\left[1-\left(1-k_{0}\right)Iv\left(P\right)\right]}+G=0$$

In Equation (4), V is the growth rate of the dendrite tip, m·s^−1^; Γ is the Gibbs–Thompson coefficient, m·K; P is the Peclet number of solute diffusion; D is the diffusion coefficient of solute element in liquid, m^2^·s^−1^; m is the liquidus slope, K·(wt.%)^−1^; C_0_ is the initial concentration of solute elements, wt.%; k_0_ is the distribution coefficient; Iv(P) is the Ivantsov function; and G is the temperature gradient, K·m^−1^. Ivantsov function is the steady state diffusion solution of dendrite tip obtained strictly from mathematics by mathematician Ivantsov on the basis of assuming that the solid–liquid interface is isothermal or isoconcentration parabola. Since the temperature gradient has little effect on the growth rate of the dendrite tip, the value of G is set to zero.

In order to increase the universality of the above KGT model in solidification simulation [25], the KGT model is extended in multiple components, and Equations (5)–(7) are obtained:

(5) $$c_{0}=\sum c_{i}$$

(6) $$m=\sum \frac{m_{i}c_{i}}{c_{0}}$$

(7) $$k=\sum \frac{m_{i}c_{i}k_{i}}{mc_{0}}$$

In Equations (5)–(7), ci is the mass fraction of the alloy element; mi is the liquidus slope of the alloy element; and ki is the solute equilibrium partition coefficient of the alloying element.

According to the theory of metal solidification [26], there is a supercooled melt at the dendrite front during grain growth, and the dendrite growth is affected by dynamic supercooling, compositive supercooling, thermal supercooling, curvature supercooling and other factors, as shown in Equation (8):

(8) $$\Delta T=\Delta T_{c}+\Delta T_{t}+\Delta T_{k}+\Delta T_{r}$$

In Equation (8), ΔT_c_ is the component subcooling degree, K; ΔT_t_ is the degree of thermodynamic undercooling, K; ΔT_r_ is the curvature subcooling degree of solid–liquid interface, K; and ΔT_k_ is growth dynamic subcooling, K. For most metals, the latter three values are small and negligible compared to the subcooling ΔT_c_.

To simplify the calculation, Rappaz and Kurz give a simplified relationship between dendrite tip growth rate and subcooling degree based on the KGT model, and the following polynomial fitting Equation (9) is obtained.

(9) $$v\left(\Delta T\right)=a_{2}\Delta T^{2}+a_{3}\Delta T^{2}$$

In Equation (9), a_2_ and a_3_ are polynomial coefficients, respectively, which are alloy-related constants, m·s^−1^·K^−2^; and ΔT is the degree of subcooling of the dendrite tip, K.

### 2.3. Model Parameters and Boundary Conditions

In this paper, the three-dimensional modeling software SolidWorks 2019 was used to model the casting billet. The overall dimension cross-section of the casting billet was 200 mm × 200 mm. The established stereoscopic model of the casting billet was imported into the ProCAST software 17.5 to grid the model with tetrahedral mesh, and the total number of grids of the model was 1372037.

In this paper, the Scheil model was selected for simulation. It is a mathematical model used to simulate the solidification process of alloys. Named after the German metallurgist Theodoor Scheil, the model is used to describe the changes in the composition of the solid and liquid phases of alloys during solidification.

To limit the computational time, the model relies on the following assumptions:

(1) The solidification shrinkage was not taken into account;
(2) The self-nucleation was not taken into consideration;
(3) The model assumes that the solidification process was carried out under equilibrium conditions, and each solid phase and liquid phase in the alloy were formed under their equilibrium conditions;
(4) The model ignores the reverse reaction between the solid phase and liquid phase, and the formed solid phase no longer reacts with the liquid phase;
(5) The simulation assumes that the elements in the alloy are ideally mixed, and the effect of element diffusion on the solidification structure was ignored.

In this study, the billet elements from a steel mill were imported into ProCAST software 17.5, and c_i_, m_i_, k_i_ and other values required for simulation could be calculated, as shown in Table 1. The G-T coefficient of 20# steel is 2 × 10^−7^ m·K, and the growth kinetic parameter a_2_ = 1.798 × 10^−6^ m·s^−1^·k^−2^. a_3_ = 3.529 × 10^−6^ m·s^−1^·K^−2^.

**Figure 1:**
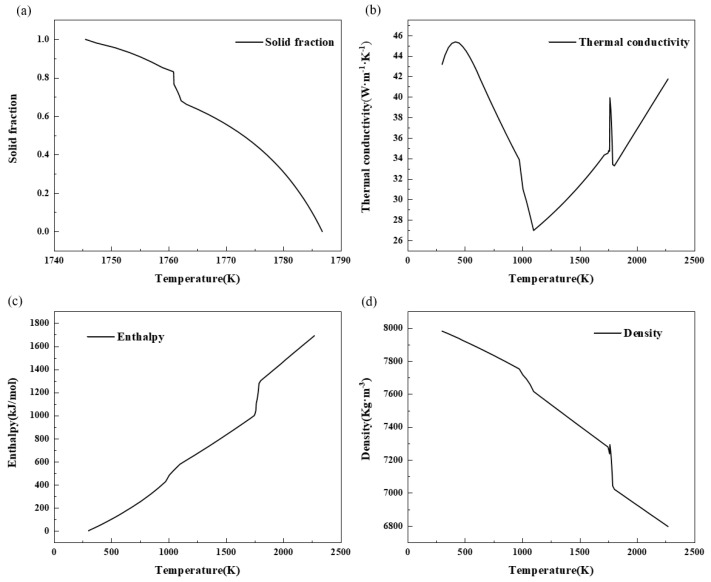
Thermophysical property parameters.

The thermal physical parameters used in the simulation process include enthalpy change, density thermal conductivity, solid phase ratio, etc., as shown in Figure 1. The liquidus is 1786 K, and the solid phase is 1751 K.

Considering the efficiency and accuracy of numerical simulation, appropriate simplification was conducted in the process of numerical simulation. The filling process was ignored in the simulation, and the initial state of the model was assumed to be full of melt. Specific nucleation simulation parameters in the simulation process are shown in Table 2. The nucleation parameters adopted in the simulation mainly refer to the values provided by ESI Company, and the influence of electromagnetic stirring on nucleation parameters is considered [27]; where n_v, max_ is the maximum volume nucleation density; ΔT_v, max_ is the maximum supercooling of volume nucleation, ΔT_v, σ_ is the maximum supercooling standard deviation of volume nucleation. n_s, max_ is the maximum surface nucleation density, ΔT_s, max_ is the maximum supercooling of surface nucleation, ΔT_s, σ_ is the maximum supercooling standard deviation of surface nucleation. The values in Table 2 are derived from the experience of searching ESI companies and publishing-related papers [24,25,26]. These references are all publicly published books. When the values were cited in the manuscript, we significantly added reference numbers with full source information. Therefore, under the terms of the Creative Commons CC BY license, we believe that these references could be cited without permission.

## 3. Experimental Implementation

**Figure 2:**
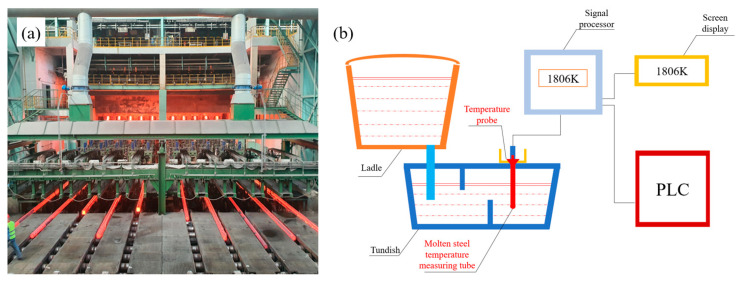
Billet production: (**a**) Continuous casting production site; (**b**) Continuous temperature measuring equipment.

On the basis of simulation, experiments were carried out using a small billet continuous casting machine in a steel plant. The main parameter of the continuous casting machine is a 10-flow full arc square billet continuous casting machine, and the main parameters of the cooling system are the use of segmented water cooling as well as aerosol cooling. The main composition of the billet is 20# steel. Figure 2a shows the continuous casting production site, and Figure 2b shows the superheat continuous temperature measurement equipment, and superheat measurement using BCT-V-A continuous temperature measurement device. This device is manufactured in China Shenyang Taihe Metallurgical Measurement and Control Technology Co., Ltd. (Shenyang, China). The cast billet was processed by grinding machine and pickling to treat the low-power structure. We used 30% hydrochloric acid and water to mix 1:1. We heated the acid solution to 328 K and let the cast billet stand for 5–10 min to obtain the low-power structure, then we observed and measured the equiaxed crystal region.

## 4. Results and Discussion

### 4.1. Simulation Verification

**Figure 3:**
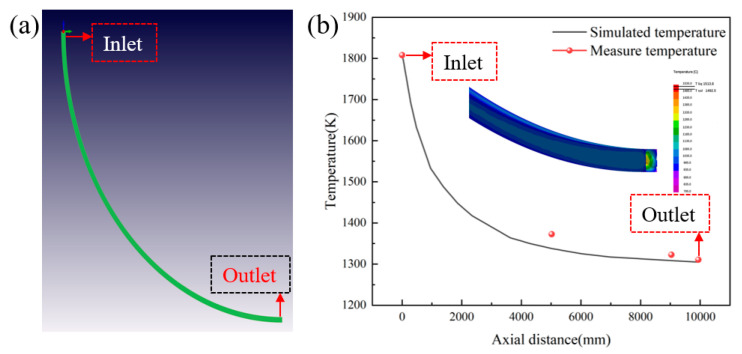
Comparison between simulated surface temperature and measured value: (**a**) Continuous casting model; (**b**) Comparison of test and simulation results.

A full-size model of continuous casting was established, with a cross-section of 200 mm × 200 mm and an arc radius of 10,000 mm. The wall heat transfer parameters were adjusted, and the inlet temperature was set to 1806 K, that is, the superheat was 20 K. The surface temperature was simulated, and the actual temperature was measured with an infrared temperature gun on the basis of the simulation. The measured data were compared with the simulated data, as shown in Figure 3.

From Figure 3, the simulation data match at both ends. At the axial distance of 5500 mm, the measured temperature is 1373 K, while the simulated temperature is 1338 K; the reason for the error is in the measurement using the infrared thermometer, which measures the surface fluctuations and the temperature difference is large. The measured temperature at the axial distance of 10,000 mm is 1311 K, and the simulated temperature here is 1305 K; the relative error is 0.58%, and it can be considered that the model verification is accurate. After simulation verification, the calculation results of the final 50 mm of the whole process model were intercepted for analysis.

### 4.2. Simulation of Solidification Structure Evolution with Different Superheat

Columnar crystal evolution is a form of crystal growth during solidification, usually along a certain direction or axial growth, forming columnar grains. Temperature gradient and solidification rate are the key factors affecting the solidification structure and evolution of columnar crystals. Smaller temperature gradients and slower solidification rates are more conducive to the formation of equiaxed crystals. Therefore, in this study, by controlling the superheat parameters of the continuous casting process, the growth of columnar crystals and the transformation of columnar crystals to equiaxed crystals in continuous casting steel were simulated.

**Figure 4:**
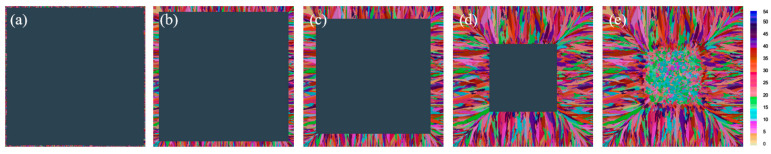
Superheat 60 K billet solidification process: (**a**) 1 s; (**b**) 42 s; (**c**) 92 s; (**d**) 202 s; (**e**) 252 s.

**Figure 5:**
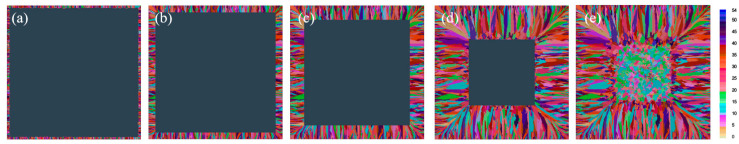
Superheat 40 K billet solidification process: (**a**) 1 s; (**b**) 42 s; (**c**) 92 s; (**d**) 202 s; (**e**) 250 s.

**Figure 6:**
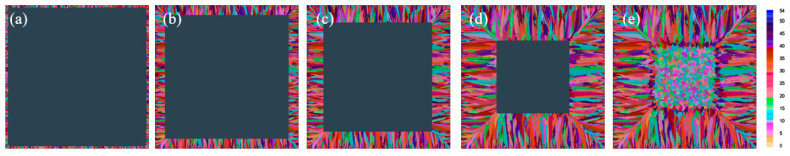
Superheat 20 K billet solidification process: (**a**) 1 s; (**b**) 42 s; (**c**) 92 s; (**d**) 202 s; (**e**) 245 s.

Low-temperature casting can reduce the internal and external temperature gradient of the billet and inhibit the growth of columnar crystals. This study simulated the solidification structure of the casting billet by changing the superheat [28,29]. In order to simplify the calculation of the simulation, the simulated temperature calculated in the model validation was used to simulate the model with a thickness of 50 mm. Figure 4 is the schematic diagram of the cross-sectional solidification process of the billet when the superheat is 60 K. Figure 5 is the schematic diagram of the cross-sectional solidification process of the billet when the superheat is 40 K. Figure 6 is the schematic diagram of the cross-sectional solidification process of the billet when the superheat is 20 K. 

From Figure 4, at the 1 s mark, the presence of substantial heat exchange at the wall initiates rapid solidification, giving rise to the formation of an excitatory cooling layer. This process results in the development of a thin, finely crystallized zone. By the 42 s mark, columnar crystal growth becomes evident in Figure 4b, originating from the fine crystal zone. Notably, the orientation of columnar crystal growth exhibits inconsistency, attributed to the anisotropy arising from inconsistent cooling intensity along the heat flow direction. As time progresses to 92 s, the columnar crystals reach full growth. Subsequently, at 202 s, a significant transformation known as the columnar-to-equiaxed transition (CET) occurs at the conclusion of columnar crystal growth. This transition marks the shift from columnar crystal morphology to equiaxed crystal formation. Finally, at the 252 s mark, the cast billet undergoes complete solidification, signifying the culmination of the observed stages in the continuous casting process. These temporal landmarks, elucidated through the sequential analysis of Figure 4, offer crucial insights into the dynamic evolution of microstructural features during the solidification process. The observed phenomena, including the development of the excitatory cooling layer, fine crystal zone, and the subsequent CET transformation, contribute to a comprehensive understanding of the intricate interactions taking place in the continuous casting of billets.

From Figure 5, when the time is 1 s, the grain growth of the section is compared and the chilling layer of the wall is fully grown at the same time. When the time is 42 s, the columnar crystal starts to grow, and the growth rate of the columnar crystal will be different due to the different superheat. When the time is 92 s, the cylindrical crystal in the corner begins to grow to the center position, which promotes the formation of equiaxed crystals to a certain extent. When the time is 202 s, the columnar crystals have grown completely, and CET transformation is carried out at the apex of the columnar crystals. Compared with the superheat of 60 K, the transition trend of columnar crystals to equiaxial crystals is different due to the temperature difference at the same time point. When the time is 250 s, the casting billet is completely solidified, and there is a tendency to expand in the central equiaxed crystal region when the superheat is 60 K, and the complete solidification time is reduced by 2 s when the superheat is 60 K, indicating that the decrease of superheat also slows down the growth rate of the solidified structure cylindrical crystals and equiaxed crystals.

**Figure 7:**
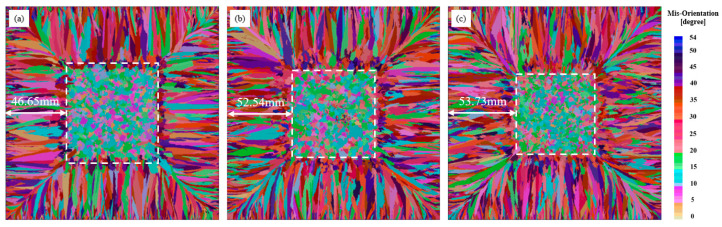
Simulation results of different superheat degrees on solidification organization: (**a**) Superheat 20 K; (**b**) Superheat 40 K; (**c**) Superheat 60 K.

From Figure 6, when the time is 1 s, compared with the superheat of 60 K and 40 K, the chill layer of 20 K is fully grown, and a fine crystal zone of about 5 mm is formed on the wall. When the time is 42, the growth rate of columnar crystals is faster because of the low superheat. When the time is 92 s, the growth of corner columnar crystals accelerates the growth of wall columnar crystals to the center position. Compared with the superheat conditions of 60 K and 40 K, the maximum distance between the growth end of columnar crystals and the wall is 26 mm. When the time is 202 s, the columnar crystal achieves complete growth and CET transition occurs at the end of columnar crystal growth. When the time is 245 s, the complete solidification time is reduced by 7 s compared with the casting billet with 60 K superheat, 5 s compared with the casting billet with 40 K superheat, and the central equiaxed crystal rate is increased, the superheat is further reduced, the equiaxed crystal area is expanded and the performance of the casting billet is improved. Figure 7 shows the dendrite growth of solidified tissue at the section of casting billet with superheat of 20 K, 40 K and 60 K.

**Figure 8:**
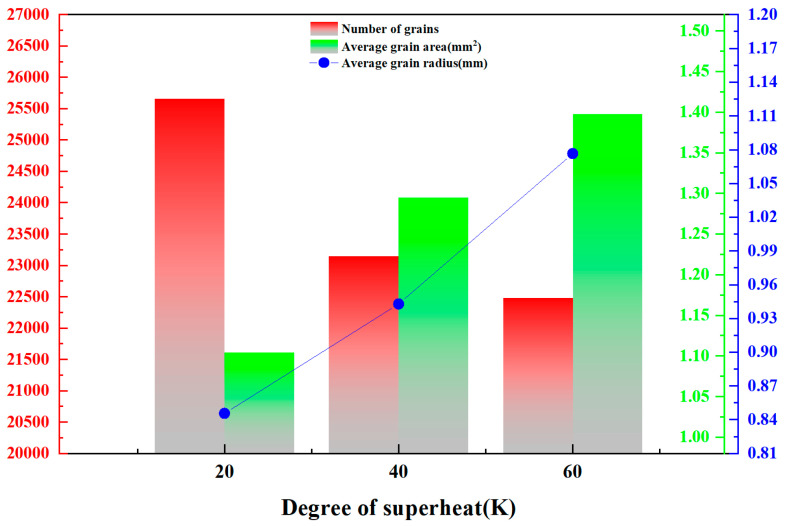
Statistics on the effect of different superheat degrees on the size of equiaxed crystals.

From Figure 7, the solidification organization is divided into three regions, the surface fine crystal region, the columnar crystal region and the central equiaxed crystal region. The evolution rule is that the fine crystal zone on the surface of the ingot gradually develops into a coarse columnar crystal zone along the radial direction, while the central part of the ingot is the equiaxed crystal zone, and the simulated solidification tissue evolution is in accordance with the solidification theory. It is obvious from the solidification tissue growth state that the size of columnar crystals becomes larger and larger as the superheat increases, and the original growth area of equiaxed crystals is occupied by columnar crystals, while the growth area of equiaxed crystals decreases in scope and the size of equiaxed crystals also increases slightly. In order to analyze the simulation results in detail, the isometric crystal size measurements were performed using ImageJ 1.8.0 software. Table 3 shows the calculation results about the number of grains, etc., under different superheat degrees, and Figure 8 shows the effect of different superheat degrees on the isometric crystal size.

From Table 3, the percentage of equiaxed crystal zone decreases from 53.35% to 46.27% when the superheat degree increases from 20 K to 60 K. With the increase of superheat degree, the temperature gradient in the liquid metal increases, which is not conducive to the formation of nuclei inside the melt, resulting in the decrease of the percentage of equiaxed crystal zone. With the increase of superheat degree, a larger temperature gradient exists in the solid–liquid phase dendrite front, which promotes the growth of columnar crystals and expands the proportion of columnar crystals. From Figure 8, the red number represents the number of grains, the green number represents the average grain area, and the blue number represents the average grain radius. The number of grains decreases when the superheat degree increases from 20 K to 60 K, the number of grains decreases from the maximum value of 25,662 to 22,476, the average grain area increases from 1.104 mm^2^ to 1.398 mm^2^, and the average grain size increases from 0.84556 mm to 1.07653 mm, which reduces the heat transfer efficiency of the steel due to the increase of superheat degree. In this case, the degree of nucleation supercooling is not sufficient to provide isometric nucleation growth force, resulting in columnar crystal growth and reduction of isometric crystal area. On the other hand, the increase in temperature causes the solidified columnar crystals to remelt and inhibits the growth of equiaxed crystals.

### 4.3. Experiment of Influence of Different Superheat on Solidification Structure

**Figure 9:**
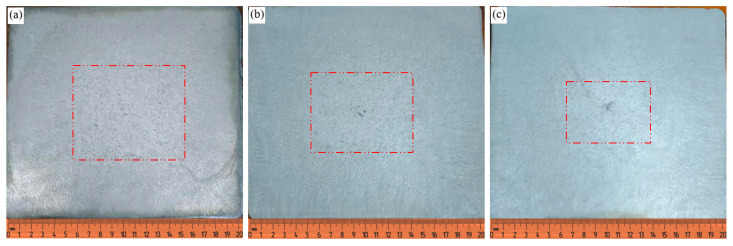
Test results of cast billets at different superheat levels: (**a**) Superheat 20 K; (**b**) Superheat 40 K; (**c**) Superheat 60 K.

**Figure 10:**
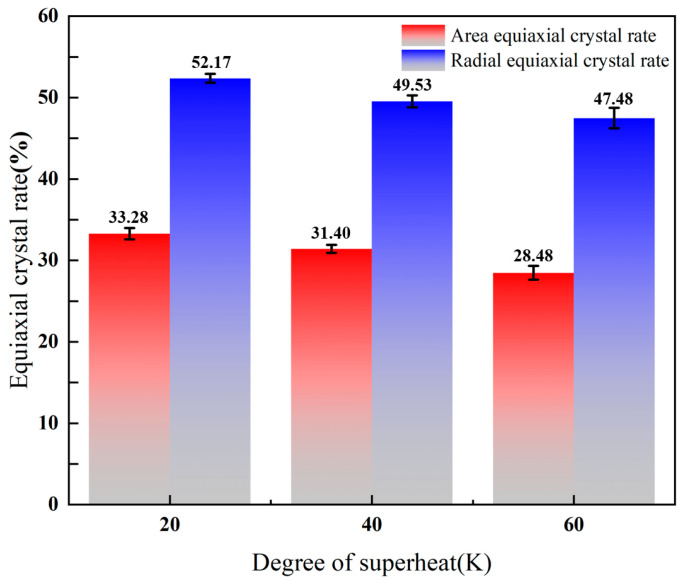
The influence of superheat on the area equiaxed crystal rate and radial equiaxed crystal rate of casting billet.

Figure 7 shows the pictures of the low power structure of a 200 mm × 200 mm small billet at superheat degrees of 20 K, 40 K and 60 K. The low power structure is divided into equiaxed crystal areas using ImageJ 1.8.0 software, and the area divided by the red line in Figure 9 is the equiaxed crystal area. The comparison of the low power structure at superheat 20 K with the simulation results is shown in Figure 10.

From Figure 9, it can be seen that under different superheat conditions, there is a big difference in the growth morphology of the cast billet low times, when the superheat is lower, the difference of the melt temperature gradient is smaller, the columnar crystal grows more slowly in the solidification front, and the supercooled liquid zone in front of the columnar crystal tip also widens, which inhibits the growth of columnar crystal and increases the proportion of equiaxed crystal zone. In Figure 9b,c, it is obvious that high superheat degree casting will cause central shrinkage defects in the cast billet.

**Figure 11:**
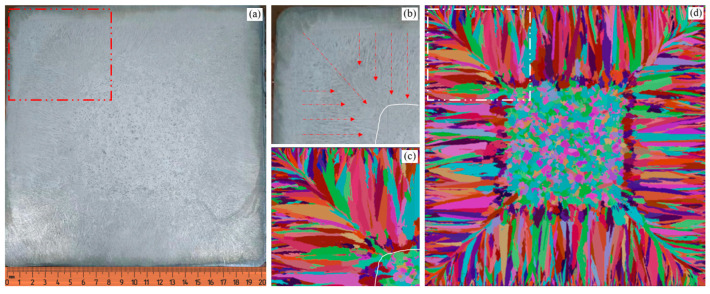
Comparison of test and simulation results under 20 K superheat condition: (**a**) Test results; (**b**) Test results corner; (**c**) Corners of simulation results; (**d**) Simulation results.

**Figure 12:**
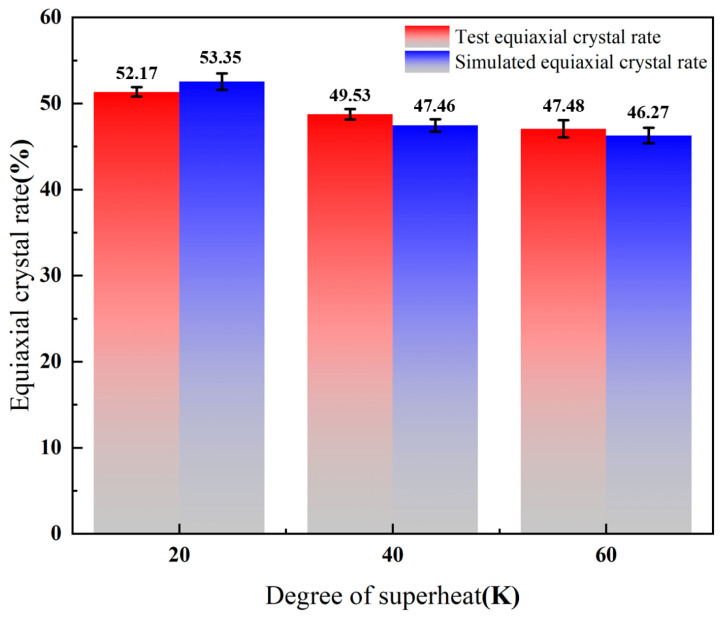
The effect of superheat on the equiaxed crystal rate of casting billet is compared between simulation results and test results.

From Figure 10, with the increase of superheat, the area equiaxed crystal rate decreases from 33.28% to 28.48%, and the radial equiaxed crystal rate decreases from 52.17% to 47.48%. Therefore, the equiaxed crystal rate decreases with the increase of superheat. The change of superheat mainly affects the growth of columnar crystals and the formation of equiaxed crystal regions. In the case of low superheat, the grain growth may be more random, resulting in a more uniform grain orientation distribution in the final billet, leading to a more dense equiaxed grain [30]. At the same time, a lower superheat may produce a relatively uniform texture. The formation of texture is closely related to the orientation of grain, which has a significant effect on the anisotropy and mechanical properties of the material [31]. The effect of superheat on crystal direction is realized by adjusting the mode and orientation of grain growth. In the continuous casting process, the orientation distribution of the crystals during solidification can be adjusted by controlling the superheat, thus affecting the microstructure and macroscopic properties of the final casting billet [32]. Figure 11 shows the effect of different superheat degrees on the equiaxed crystal rate of cast billets. Figure 12 shows the comparison between the simulated results and the test results on the equiaxed crystal rate of the casting billet under different superheat conditions.

From Figure 11, the red arrow in the figure indicates the growth direction of the columnar crystal, and the edge area is enlarged. We combine Figure 11 and Figure 12 for analysis, it can be seen that the solidification organization of the small billet tends to be consistent with the simulation results under the same superheat degree, which confirms the feasibility of the simulation. When the superheat is 20 K, the simulated equiaxed crystal rate reaches the highest value, which is 53.35%, and the standard deviation is 0.55%. When the superheat is 40 K, the simulated equiaxed crystal rate decreases to 47.46% and the standard deviation is 0.61%. When the superheat is 60 K, the simulated equiaxed crystal rate reaches the lowest value, which is 46.27%, and the standard deviation is 1.01%. It can be seen that billet defects such as shrinkage are prone to occur at high superheat, so a lower superheat should be controlled in practice to expand the area of the isometric crystal zone and improve the quality of the billet.

## 5. Conclusions

(1) The simulation results of solidification microstructure evolution during the solidification process of casting billet with different superheats indicate that increasing superheat can inhibit the growth of equiaxed crystals and reduce the size of equiaxed crystals. The lower the superheat, the lower the edge temperature gradient, and the slower the solidification rate of the billet, which is more conducive to the growth of the medium axis of the billet. When the superheat increases from 20 K to 60 K, the proportion of the equiaxed crystal region decreases from 53.35% to 46.27%. This relationship reflects the influence of superheat on the solidification process of casting billet.
(2) The experimental study of the effect of different superheats on the solidification structure of the casting billet shows that under the same conditions, the difference in equiaxed crystal rate is not significant between the simulated results and the field test results. The proportion of the equiaxed crystal region at low magnification shows that the equiaxed crystal region decreases and the columnar crystal region increases when the superheat is increased from 20 K to 60 K. Under the test condition that the superheat is increased from 20 K to 60 K, the equiaxed crystal rate decreases from 52.17% to 47.48%. When the simulated superheat increases from 20 K to 60 K, the equiaxed crystal rate decreases from 53.35% to 46.27%, indicating that the effect of different superheats on the solidification structure is consistent with the simulated results.

## Figures and Tables

**Table 1:** Simulation calculation parameter.

Element	Content (wt.%)	k	m	D/m^2^·s^−1^
C	0.200	0.170784	−82.6456	3 × 10^−9^
Si	0.270	0.594312	−16.3372	3 × 10^−9^
Mn	0.500	0.710554	−5.22204	3 × 10^−9^
P	0.035	0.310875	−27.0198	3 × 10^−9^
S	0.035	0.036319	−39.9102	3 × 10^−9^
Cr	0.010	0.884948	−2.20964	3 × 10^−9^
Ni	0.010	0.818543	−3.33588	3 × 10^−9^
Cu	0.010	0.827207	−3.48933	3 × 10^−9^

**Table 2:** Nucleation simulation parameters.

Nucleation Parameter	n_v_, _max_/K	ΔT_v_, _max_/K	ΔT_v_, _σ_/K	n_s_, _max_/K	ΔT_s_, _max_/K	ΔT_s_, _σ_/K
Value	1 × 10^9^	10	1	3 × 10^7^	10	1

**Table 3:** Calculated the number of grains at different superheats.

Statistical Items	Superheat 20 K	Superheat 40 K	Superheat 60 K
Number of grains	25,662	23,146	22,476
Average grain area/mm^2^	1.104	1.295	1.398
Average grain radius/mm	0.84556	0.94281	1.07653
Percentage of radial equiaxed crystal region/%	53.35	47.46	46.27

## Data Availability

The data presented in this study are available on request from the corresponding author.
